# Cohort dominance rank and “robbing and bartering” among subadult male long-tailed macaques at Uluwatu, Bali

**DOI:** 10.1038/s41598-022-11776-7

**Published:** 2022-05-13

**Authors:** Jeffrey V. Peterson, Agustín Fuentes, I Nengah Wandia

**Affiliations:** 1 Arts and Sciences Division, Rend Lake College, 468 N. Ken Gray Parkway, Ina, IL 62846 USA; 2 Department of Anthropology, Princeton University, Princeton, USA; 3 Department of Veterinary Sciences, Udayana University, Denpasar, Indonesia

**Keywords:** Behavioural ecology, Animal behaviour

## Abstract

Robbing and bartering is a habitual behavior among free-ranging long-tailed macaques (*Macaca fascicularis*) at a single site in Bali, Indonesia. The behavior consists of three main elements: (1) a macaque takes an item from a human; (2) the macaque maintains possession of the item; then (3) the macaque releases or hands off the item after accepting a food offer from a human. In this paper, we analyze data on individual variation in robbing and bartering among subadult males in relation to dominance rank. Using focal animal sampling we collected 197 observation hours of data on 13 subadult males from two groups (6 from Celagi; 7 from Riting) at the Uluwatu temple site from May 2017 to March 2018, recording 44 exchanges of items for food from 92 total robberies following 176 total attempts. We also measured dominance rank using interaction data from our focal animals. Dominance rank was strongly positively correlated with robbery efficiency in Riting, but not Celagi, meaning that more dominant Riting subadult males exhibited fewer overall robbery attempts per successful robbery. We suggest the observed variation in robbing and bartering practices indicates there are crucial, yet still unexplored, social factors at play for individual robbing and bartering decisions.

## Introduction

Robbing and bartering is a behavioral pattern in which free-ranging nonhuman primates spontaneously steal an object from a human (usually tourists), and then hold onto that object until that or another human solicits an exchange by offering food^1–3^. This behavior has been systematically studied in the Uluwatu long-tailed macaque (*Macaca fascicularis*) population in Bali, Indonesia^1,2,4^, but also reported in other populations in Bali^5–7^^: 141^. Outside of Bali, object stealing (including sometimes, but not always, subsequent exchange for food) has been observed in rhesus macaques (*Macaca mulatta*)^8,9^ and Tibetan macaques (*Macaca thibetanus*)^10^, and is anecdotally reported for macaque populations across South and Southeast Asia.

Spontaneous robbing and bartering by the long-tailed macaques at the Uluwatu temple in Bali, Indonesia combines multiple behaviors into a single behavioral sequence. Previous reports on robbing and bartering in this population establish two parts to the behavior, the robbery and the exchange, and model it primarily as an economic activity^1,2,4^. We suggest this behavior also involves an important third phase in the sequence: the period of item possession between the robbery and the exchange. Therefore, we characterize the robbing and bartering sequence as follows: (1) robbing the item; (2) holding and/or manipulating the item before the potential exchange; and (3) exchanging the item.

The robbing and bartering sequence in the Uluwatu long-tailed macaque population may be a behavioral tradition (i.e., cultural behavior)^11^, given evidence of intergroup variation in robbing and bartering patterns^1,2^, that individuals rob more frequently after observing a demonstrator robbing^4^, and evidence for persistence across generations^1,4^ and socially mediated, age-based learning^2^. Groups spending more time in tourist zones, close to humans, and consuming provisioned food were found to be more likely to rob items than groups that did not^1^. Despite these differences in robbery occurrence, there were no observed differences in robbing or bartering success rates between groups^1^.

### Token exchange and dominance rank position

Social context matters for exchange behavior among nonhuman primates^12^. For instance, researchers found dominance rank can influence learning how to exchange and subsequently participating in exchanges^13^. Naïve higher-ranking capuchins (*Cebus apella*) learned the token exchange protocol in a social context, and exchanged higher-valued tokens more frequently, whereas lower ranking individuals were unable to learn the exchange in a social context or exhibit the exchange in social or solitary contexts^13^. This outcome is interesting, given the tendency for innovation of cultural behaviors to occur in younger (and potentially lower ranking) individuals and then become transmitted to their close kin and other younger group members ^14^.

If rank effects are important in exchange behaviors in captivity, they may be particularly salient for free-ranging populations due to the more dynamic and vital impact social rank and context can have on individual wellness and group stability. For many nonhuman primate species, stable rank hierarchies reduce stress in dominant individuals but not subordinates^15^. Thus, in captivity, lower ranking individuals may be reticent to exchange in social situations due to coping mechanisms involving avoiding dominants, which can be more difficult to accomplish in captive settings^13,15^. However, free-ranging populations do not have the structural and spatial constraints of captive ones, thus the potential for a wider range of responses or patterns in negotiating exchanges is present^12^. Characterizing and understanding individual variation in spontaneous robbing and bartering practices in relation to dominance status can therefore contribute to our understanding of robbing and bartering among the Uluwatu macaques in Bali.

### Study goals and aims

All robbing and bartering contexts and outcomes are different and multivariate. We focused on the effect of dominance rank in 13 individual monkeys belonging to a single age and sex class, subadult males, and analyzed a relatively small sample of events. Our first aim is to establish a set of metrics that future work on robbing and bartering can use to structure research questions and hypotheses. This step contributes to a crucial “natural history” stage expanding baseline knowledge of robbing and bartering^16^, which has only recently come under systematic study^1,4^. Therefore, we ask first, is there variation in robbing and bartering parameters that we can observe at group or individual levels? We examined the following parameters to assess this question: (1a) total robbery events; (1b) robbery efficiency (defined below); (1c) Exchange Outcome Index (defined below); (1d) stolen item type; and (1e) type of food reward received.

Our second aim is to assess the potential influence of dominance rank on the robbing and bartering metrics established above, given the documented rank effects on exchange behavior in captive settings. Here, we assess the relationship between dominance rank and exchange behavior in a free-ranging population by asking: Are patterns of variation in robbing and bartering among subadult males associated with dominance rank?

We answer these questions with behavioral data collected on subadult male long-tailed macaques in two focal groups at Uluwatu. We focus on subadult males for two main reasons. First, these data on robbing and bartering were collected during a multi-sited study investigating social behavior within this life-history stage^17^. Second, the potential importance of robbing and bartering as a strategy for subadult males is supported by a previous study finding that they exhibited higher rates of robberies per focal animal than any other age-sex class in that study group^1^.

## Methods

### Study site

We conducted this research at the Uluwatu temple site in Bali, Indonesia. Uluwatu is located on the Island’s southern coast, in the Badung Regency. The temple at Uluwatu is a Pura Luhur, which is a significant temple for Balinese Hindus across the island and is therefore visited regularly for significant regional, community, family, and household rituals by Balinese people from different regions throughout the year^18^. During the period of data collection hundreds of tourists also visit the Uluwatu temple each day. The temple sits on top of a promontory cliff edge, with walking paths in front of it that continue in loops to the North and South. These looping pathways surround scrub forests, which the macaques frequently inhabit but the humans rarely enter.

In 2017–2018 there were five macaque groups at Uluwatu, which ranged throughout the temple complex area, and beyond. All groups are provisioned daily with a mixed diet of corn, cucumbers, and bananas by temple staff members. The two groups included in this research are the Celagi and Riting groups. We selected these groups because they previously exhibited significant differences in robbing frequencies whereby Riting was observed exhibiting robbing and bartering more frequently than Celagi^1^. Furthermore, both groups include the same highly trafficked tourist areas in their overlapping home ranges relative to the other groups at Uluwatu, theoretically minimizing between group differences in the contexts of human interaction^1,19^.

### Data collection

JVP collected data from May, 2017 to March, 2018 totaling 197 focal observation hours on all 13 subadult males in Celagi and Riting that were identified in May–June 2017. Subadult male long-tailed macaques exhibit characteristic patterns of incomplete canine eruption, sex organ development, and body size growth, which achieves a maximum of 80% of total adult size^18^. Mean sampling effort per individual was 15.2 hours (h), with a range of 1.75 h, totaling 102.75 h for Riting and 94.75 h for Celagi. The data collection protocol consisted of focal-animal sampling and instantaneous scan sampling^20^ on all six subadult males in the Celagi group, and all seven subadult males in the Riting group. Focal follows were 15 minutes in length. Sampling effort per individual is presented in Table 1. A random number generator determined the order of focal follows each morning. In the event a target focal animal could not be located within 10 minutes of locating the group, the next in line was located and observed. Data presented here come from focal animal sampling records of state and event behaviors. Relevant event behaviors consist of agonistic gestures used for calculating dominance relationships, including the target, or interaction partner, of all communicative event behaviors and the time of its occurrence. All changes in the focal animal’s state behavior were noted, recording the time of the change to the minute.

**Table 1:** Focal Subadult male long-tailed macaques in Celagi and Riting at Uluwatu, Bali, Indonesia.

Group	Individual	Sampling effort (h)
Celagi	Chip	14.5
	Fizz	15.5
	Gru	15.5
	Hemingway	16.25
	Minion	15.25
	Spot	15.5
Riting	Babyface	15
	Bilbo	15
	Chaplin	15.5^a^
	Hanoman	14.5
	Ichabod	15
	Pancake	15
	Winky	15

^a^2.25 h of this total sampling effort occurred while this individual was a member of Celagi, and contributes to the reported Celagi sampling effort.

During focal samples we recorded robbing and bartering as a sequence of mixed event and state behaviors. We scored both the robbery and exchange phases as event behaviors, and the interim phase of item possession as a state behavior. We record a robbery as successful if the focal animal took an object from a human and established control of the object with their hands or teeth, and as unsuccessful if the focal animal touched the object but was not able to establish control of it. For each successful robbery we recorded the object taken. Unsuccessful robberies end the sequence, whereas successful robberies are typically followed by various forms of manipulating the object.

The robbing and bartering sequence ends with one of several event behavior exchange outcomes: (1) “Successful exchanges” consist of the focal animal receiving a food reward from a human and releasing the stolen object; (2) “forced exchanges” are when a human takes the object back without a bartering event; (3) “dropped objects” describe when the macaque loses control of the object while carrying it or otherwise locomoting, and is akin to an “accidental drop”; (4) “no exchange” includes instances of the macaque releasing the object for no reward after manipulating it; and (5) “expired observation” consists of instances in which the final result of the robbing and bartering event was unobserved in the sample period (i.e., the sample period ended while the macaque still had possession of the object). A 6th exchange outcome is “rejected exchange,” which occurs when the focal animal does not drop the stolen object after being offered, or in some cases even accepting, a food reward. The “rejected exchange” outcome is unique in that it does not end the robbing and bartering sequence because a human may have one or more exchange attempts rejected before eventually facilitating a successful exchange, or before one of the other outcomes (2–5) occurs. For each successful exchange we recorded the food item the macaques received. Food items are grouped into four categories: fruits, peanuts, eggs, and human snacks. Snacks include packaged and processed food items such as candy or chips.

### Data analysis

We grouped the broad range of stolen items into classes of general types. “Eyewear” combines eyeglasses and sunglasses, while “footwear” combines sandals and shoes. “Ornaments” includes objects attached to and/or hanging from backpacks, such as keychains, while “accessories” includes decorative objects attached to an individual’s body or clothing like bracelets and hair ties. “Electronics” covers cellular phones and tablets. “Hats” encompasses removable forms of headwear, most typically represented by baseball-style hats or sun hats. “Plastics” is an item class consisting of lighters and bottles, which may be filled with water, soda, or juice. The “unidentified” category is used for stolen items which could not be clearly observed during or after the robbing and bartering sequence.

“Robbery attempts” refers to the combined total number of successful and unsuccessful robberies. “Robbery efficiency” is a novel metric referring to the number of successful robberies divided by the total number of robbery attempts. The “Exchange Outcome Index” is calculated by dividing the number of successful exchanges by the total number of robbery attempts. We make this calculation using robbery attempts instead of successful robberies to account for total robbery effort because failed robberies still factor into an individual’s total energy expenditure toward receiving a bartered food reward and their total exposure to the risks (e.g., physical retaliation) of stealing from humans relative to achieving the desired end result of a food reward.

Social rank was measured with David’s Score, calculated using dyadic agonistic interactions. We coded “winners” of contests as those who exhibited the agonistic behavior, while “losers” were the recipients of those agonistic behaviors^21,22^. We excluded intergroup agonistic interactions in our calculations of David’s Score.

To account for potential variation in the overall patterns of interaction with humans between groups we calculated a Human Interaction Rate, which is the sum of human-directed interactions from focal animals in each group divided by the total number of observation hours on focal animals in that group.

### Statistical analysis

We ran statistical tests in SYSTAT software with a significance level set at 0.05. We used chi-square goodness-of-fit tests to assess the significance of differences in successful robberies between individuals for each group. To avoid having cells with values of zero, two focal subjects, Minion and Spot from Celagi, are excluded from this test because neither were observed making a successful robbery during the observation period. We also used chi-square goodness-of-fit tests to assess exchange outcome occurrences within each group, as well as a Fisher’s exact to test for significant differences in robbery outcomes between groups due to low expected counts in 40% of the cells. “Rejected exchange” events were not included in the analysis of robbery outcomes because they do not end the sequence and are therefore not mutually exclusive with the other robbery outcomes.

We further tested for the effect of dominance position on robbery outcomes. Due to our small sample size and the preliminary nature of this investigation, we used Spearman correlations to assess the relationship between subadult male dominance position via David’s Score and (1) robbing efficiency and (2) the Exchange Outcome Index.

### Compliance with ethical standards

This research complied with the standards and protocols for observational fieldwork with nonhuman primates and was approved by the University of Notre Dame Compliance IACUC board (protocol ID: 16-02-2932), where JVP and AF were affiliated at the time of this research. This study did not involve human subjects. This research further received a research permit from RISTEK in Indonesia (permit number: 2C21EB0881-R), and complied with local laws and customary practices in Bali.

## Results

### Research question 1: is there individual variation in robbing and bartering practices among Subadult male long-tailed macaques at Uluwatu, Bali?

Among the 13 subadult male subjects we recorded 104 total robbery attempts in Celagi, compared to Riting’s 72 attempts. Despite differences in overall robbery attempts between subadult males in each group, we recorded 46 successful robbery events in each sample (Table 2). Of the combined total 92 successful robberies from both groups, just under half of observed robberies resulted in a successful exchange outcome (44), while 31 of them ended with no exchange. Nine instances are incomplete because the sample period expired while the focal animal was still in possession of the stolen item. Stolen items were forcibly exchanged (with no bartering) five times, and on three occasions the focal animal dropped the items accidentally while locomoting (Table 3). Observation of these exchange outcomes differed significantly across the full sample (Chi-square: *X*^*2*^ = 71.696, df = 4, *P* < 0.001), as well as within each group (Celagi: *X*^*2*^ = 41.609, df = 4, *P* < 0.001; Riting: *X*^*2*^ = 30.522, df = 4, *P* < 0.001), indicating that successful exchanges are the most common outcome of successful robberies and that this pattern is not likely due to random chance. These results support analyzing robbing and bartering as a sequential behavior. Patterns of exchange outcomes did not differ significantly between groups (Fisher’s exact, *P* = 0.96), suggesting that the inter-group differences observed in the current study are negligible.

**Table 2:** Intergroup comparison of robbing and bartering patterns among Subadult Males at Uluwatu, Bali, Indonesia.

	Robberies per observation hour	Robbery efficiency	Exchanges per observation hour	Exchange Outcome Index^*a*^	Human interaction rate^*b*^
Celagi	0.49	0.44	0.24	0.22	1.85
Riting	0.45	0.64	0.2	0.29	1.61

^a^Total exchanges per robbery attempt.

^b^Human-directed interactions per observation hour.

**Table 3:** Exchange outcomes among Subadult Males at Uluwatu, Bali, Indonesia.

	Dropped objects	Expired observation	Forced exchange	No Exchange	Successful Exchange	Total
Celagi	1	4	2	15	23	46
Riting	2	5	3	16	21	46
Total	3	9	5	31	44	92

The most frequently stolen object classes were eyewear (27) and footwear (24), with total exchange percentages of 56% and 58%, respectively (see Table 4 for distribution by group). The only class of objects with a 100% exchange occurrence was electronics (e.g., phones, tablets), which were successfully exchanged for food all six times they were stolen. Individual differences in patterns of robbing and bartering were observed within each group’s sample of subadult males (Table 5: Celagi; Table 6: Riting).

**Table 4:** Robbed item class frequencies among Subadult Males at Uluwatu, Bali, Indonesia.

	Celagi		Riting		Combined Groups	
	Robbery frequency	Exchange occurrence (%)	Robbery frequency	Exchange occurrence (%)	Robbery frequency	Exchange occurrence (%)
Eyewear	13	69	14	43	27	56
Footwear	14	57	10	60	24	58
Ornaments	6	17	6	33	12	25
Plastics	2	–	7	14	9	11
Accessories	5	–	1	–	6	–
Electronics	4	100	2	100	6	100
Hats	2	50	1	–	3	33
Unidentified	0	–	5	80	5	80

**Table 5:** Individual variation in robbing and bartering for Celagi Group Subadult Males at Uluwatu, Bali, Indonesia.

Individual	Chip	Fizz	Gru	Hemingway	Minion	Spot	Significance
Attempts	4	27	57	15	1	0	
Successful robberies	2	15	20	9	0	0	Chi-square: *X*^2^ = 16.783, df = 3, *P* < 0.01
Robbery efficiency	0.5	0.56	0.35	0.6	0	0	
Robberies per observation hour	0.14	0.97	1.29	0.55	0	0	
Successful exchanges	2	5	13	3	0	0	
Exchange Outcome Index	0.5	0.19	0.23	0.2	0	0

**Table 6:** Individual variation in robbing and bartering for Riting Group Subadult Males at Uluwatu, Bali, Indonesia.

Individual	Babyface	Bilbo	Chaplin	Hanoman	Ichabod	Pancake	Winky	Significance
Attempts	4	5	6	26	8	3	20	
Successful Robberies	1	2	2	19	6	2	14	Chi-square: *X*^2^ = 46.217, df = 6, *P* < 0.01
Robbery efficiency	0.25	0.4	0.33	0.73	0.75	0.67	0.7	
Robberies per observation hour	0.07	0.13	0.13	1.31	0.4	0.13	0.93	
Successful exchanges	1	0	0	8	4	0	8	
Exchange Outcome Index	0.25	0	0	0.31	0.5	0	0.4	

**Figure 1:**
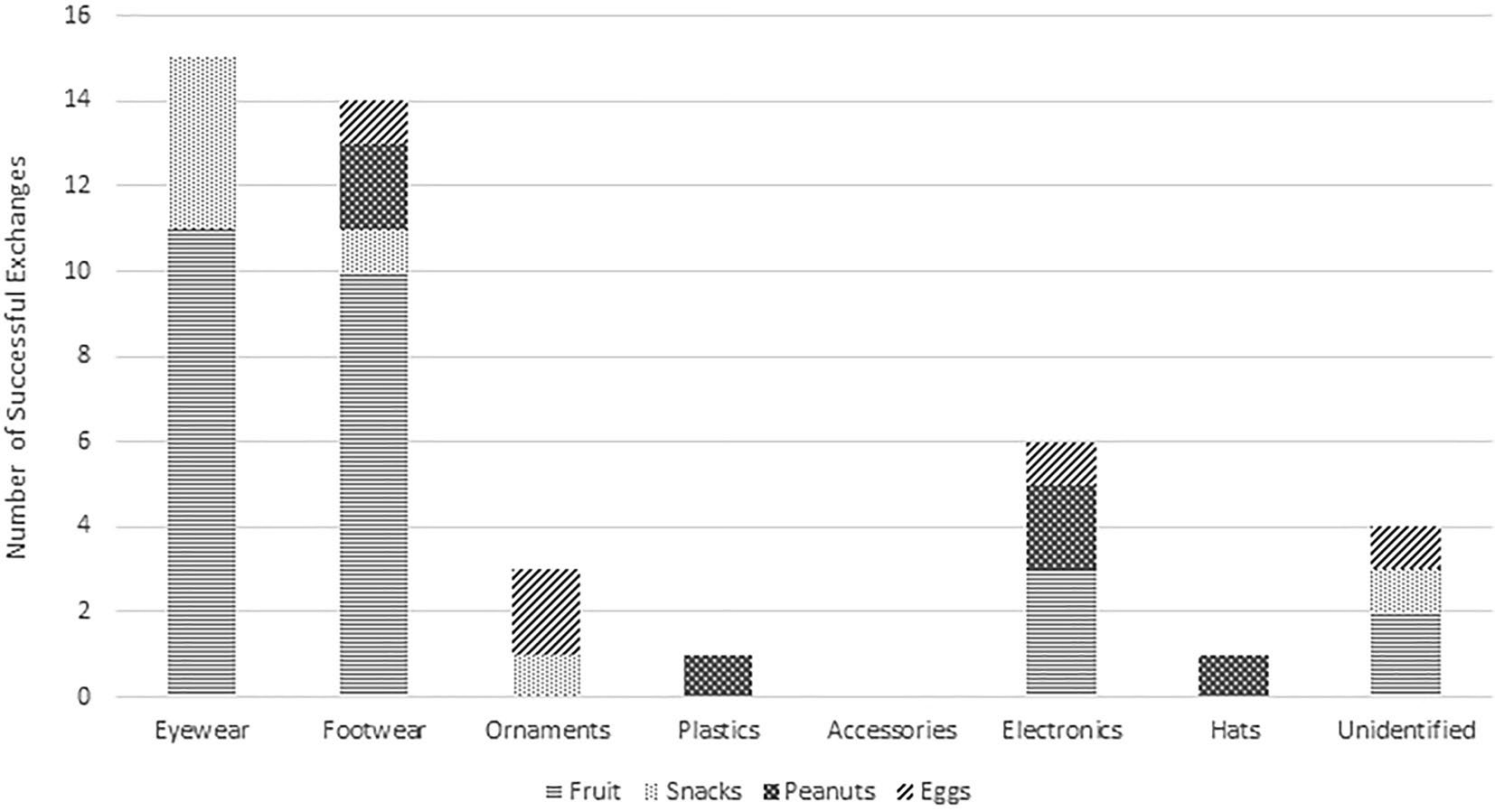
Stacked bar graph presenting rewards received for each stolen item class among subadult males at Uluwatu, Bali, Indonesia.

**Figure 2:**
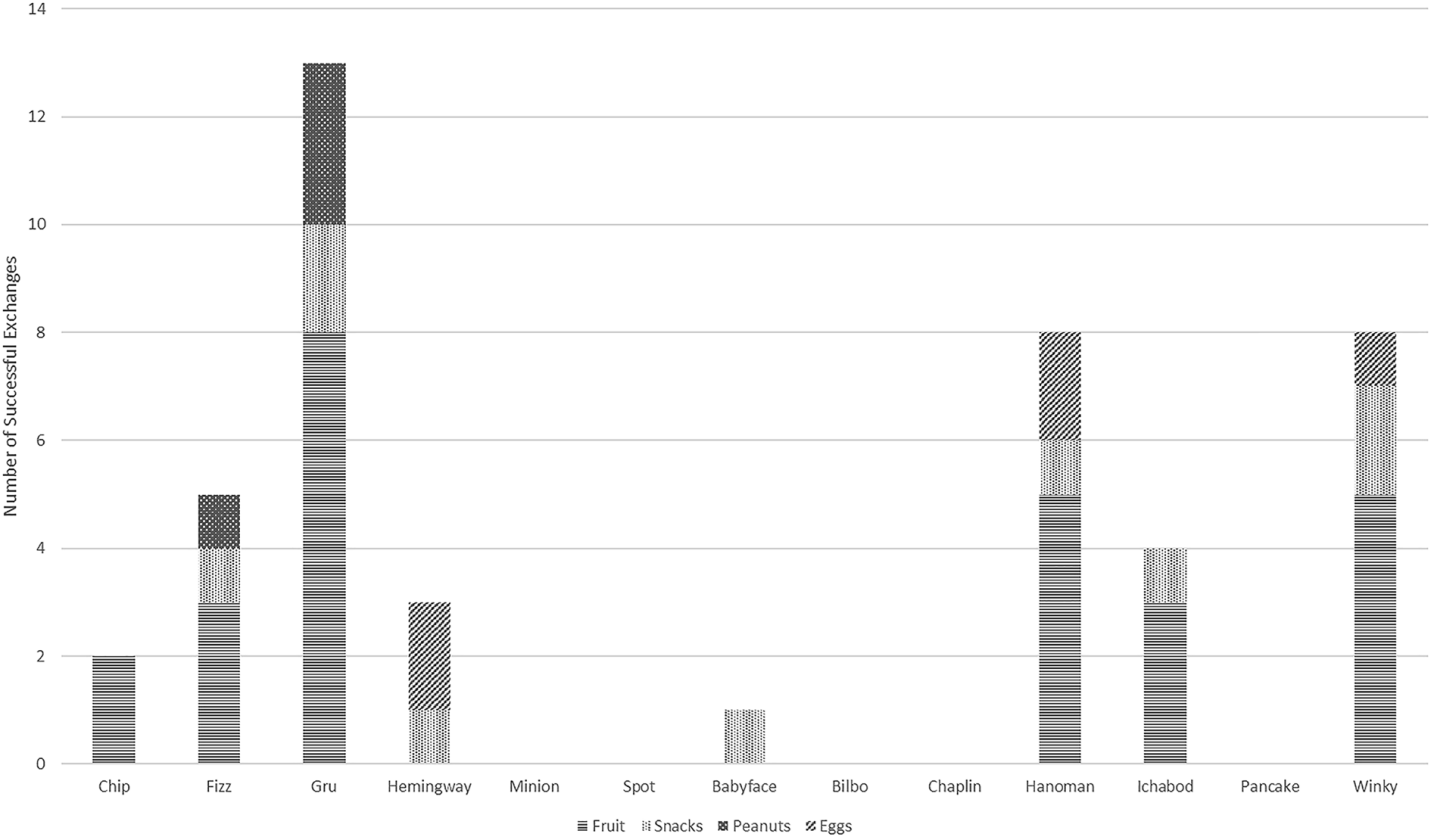
Stacked bar graph presenting rewards received for exchanging stolen items for each focal subadult male at Uluwatu, Bali, Indonesia.

Stolen items were returned in exchange for fruits more than any other class of food item. Eyewear and footwear were exchanged for fruits more often than any other stolen item class, but were stolen most frequently as well (Fig. 1). Only “ornaments”, “plastics”, and “hats” were exchanged more frequently for non-fruit food items, but the data are too few to determine a causal association. Most individuals also exchanged stolen items for fruits more than anything else (Fig. 2). However, many human staff members tend to make their initial exchange attempts with fruits, which may account for the observed preference for fruit exchanges. More data are needed to establish whether individuals prefer certain reward types.

### Research question 2: does dominance status relate to individual variation in robbing and bartering?

Dominance status for the focal subadult males is presented in Table 7. In Celagi, subadult male dominance status was weakly correlated with robbing efficiency (r_s_ = 0.3; df = 4; *P* = 0.56) and the Exchange Outcome Index (r_s_ = 0.3; df = 4; *P* = 0.56). By contrast, subadult male dominance status in Riting was strongly correlated with robbing efficiency (r_s_ = 0.9; df = 5; *P* < 0.01) and moderately correlated with the Exchange Outcome Index (r_s_ = 0.6; df = 5; *P* = 0.15). More data are needed to determine whether robbing and bartering behavior can be predicted by these or other measures of social position in a broader, more representative sample of age/sex classes.

**Table 7:** David’s score calculations among Subadult Males at Uluwatu, Bali, Indonesia.

Group	Individual	Dominance rank (David’s score)
Celagi	Chip	6.9
	Spot	6.76
	Hemingway	6.38
	Minion	5.46
	Fizz	5.28
	Gru	5.16
Riting	Hanoman	7.34
	Ichabod	6.92
	Pancake	6.38
	Winky	5.7
	Bilbo	5.6
	Babyface	5.51
	Chaplin	4.62

## Discussion

### Individual variation in robbing and bartering

Individual variation sheds light on the discrepancy in robbery efficiency found between the group samples in this study. Robbery efficiency is meant to relay the proportion of robbing successes to failures, which has implications for overall energy expenditure that could be explored in future work. Thus, looking at individual-level robbery efficiency values may indicate how successful individuals are in navigating the risks and rewards associated with exhibiting robbing and bartering. We found dominance rank to be strongly correlated with robbery efficiency among subadult males in Riting, but not Celagi. In this case, more efficient robbers tended to be the more dominant members of the subadult male cohort, who may therefore be exerting less net energy for every food exchange they receive per successful robbery compared to the more inefficient, and lower ranking, subadult males in their group. The fact that this pattern was not observed in Celagi may be relevant when we consider robbing and bartering as a cultural behavior whose practice is tied more closely to group cultural norms than to individual status markers such as dominance^4^. More work is needed to elucidate the impact of these cultural forces on observed patterns of robbing and bartering.

Celagi’s overall lower subadult male robbery efficiency came collectively from four active robbers, but essentially a single individual (Gru) brought the entire cohort’s average robbery efficiency down. Riting, on the other hand, had seven active subadult male robbers with a more even spread of robbery attempts between them. All four of Celagi’s successful robbers also participated in multiple exchanges, but only four of Riting’s seven robbers had at least one successful exchange. These results may indicate that individuals have different strategies for robbing and bartering which stem in part from their social status. For instance, low ranking individuals like Gru may go for many attempts, causing him to fail more than he succeeds at robbing. However, the sheer volume of attempts results in an overall large number of successful robberies and exchanges compared to other individuals in the study. Alternatively, some individuals may pick their robbery opportunities in only very specific situations, giving them high robbery efficiency and/or Exchange Outcome Index, but low overall return^2^. More research is needed on the range of factors affecting robbery attempt decision making to determine the extent to which personalized strategies may be utilized.

Exchange Outcome Index (defined as successful exchanges per robbery attempts) was weakly correlated with subadult male dominance status in Celagi and moderately correlated with it in Riting. These results suggest that exchanges may be even less reliant than robberies on intraspecific social processes such as dominance rank, mirroring the implications from previous findings that exchange behavior is not socially facilitated^4^. The absence of a clear social influence on exchange may be due to exchanges occurring at a greater distance from conspecifics than robberies, though this suggestion needs systematic investigation. The existence of a time gap between robberies and exchanges suggests this is a possibility, as it permits macaques to travel away from the robbery site for the exchange while carrying their stolen object. Additionally, macaques may benefit from establishing distance between themselves and conspecifics (particularly those of higher rank) to avoid conflict over the desirable food items they receive in exchange for the stolen objects (Peterson, personal observation), not unlike using distance strategies to avoid reproduction interference from dominants^23^.

Exchange Outcome Index was relatively similar between groups, but this measure is difficult to interpret at the cohort level due to the small sample size of exchanges across individuals in this study. An important caveat with respect to the Exchange Outcome Index is that not all robberies may be exhibited with the intention of a food reward. Bottles, for instance, may have their contents consumed and then be discarded. Therefore, with larger data sets it may be instructive to calculate Exchange Outcome Index for specific object classes to determine whether object-specific exchange pattens exist. For instance, robbing a water bottle, consuming its contents, and then releasing it without an exchange may not bear a meaningful relation to the Exchange Outcome Index because the macaque may intend only to drink what is inside rather than exchange it. Indeed, the only “plastic” exchanged in this study was a cigarette lighter, not any of the bottles. Thus, it is possible that the macaques have some form of flexible, or relative, concept of value that comes into play due to a stolen object’s ability to have a use-value instead of an exchange value. For instance, water bottles have a use-value making them worth stealing for the macaques because their contents can be consumed, and exchange value (potential or actual) does not factor into the equation. Furthermore, stolen bottles may not have an exchange value for the humans either, who subsequently do not seek an exchange. Therefore, the Exchange Outcome Index may be more indicative of individual robbing and bartering strategies, and the willingness of both parties to exchange, rather than individual macaque skill, cognitive capacity, or a species or population-specific economic valuation model.

The human element is an important factor in the occurrence of exchanges. For instance, footwear and eyewear were the most frequently stolen item classes in this study. Both of these categories cover a broad range of value on human economic markets that the macaques would not be able to distinguish between. In other words, the macaques do not know whether a tourist’s sunglasses are generic or branded, old or new, cheap or expensive, and therefore would not be able to gauge a tourist’s potential commitment to an exchange or the probability of receiving a food reward before a robbery. The fact that exchanges may not occur due to human disinterest in having their item returned further demonstrates that Exchange Outcome Index is less related to factors like individual macaque skill level or capabilities than our other measurement of robbery efficiency.

### Intergroup comparison of robbing and bartering at Uluwatu

Our observation of similar rates of robbing and bartering between Celagi and Riting differed from an earlier study in this population reporting more robberies per observation hour, and more time spent in the presence of humans (i.e., near footpaths and/or parking lot), in Riting compared to Celagi^1^. This change could be due in part to differences in habitat-use, whereby Celagi may be spending more time near humans throughout the current study, in a level similar to Riting, as determined by similar Human Interaction Rates (Table 2) serving as a proxy for time spent in proximity to humans.

Another facet that may be driving these differences in the robbing and bartering practices of Celagi over time (i.e., between Brotcorne et al. 2017 and the current study) is changeover in the individuals exhibiting the behaviors. The fact that the previous study included more age/sex classes in their analysis of robbing and bartering events might also be relevant; however, subadult males were key participants in the activity in the previous study as well^1^. The two most frequent robbers from this research, Gru and Hanoman, were observed in our smaller 2016 pilot study making only 0.3 robberies per observation hour apiece (Unpublished data). Robberies in that study were also tabulated using all-occurrence sampling of event behaviors during 15 minute focal follows, across 27 observation hours. Since that study, Hanoman emigrated from Celagi to Riting, while Gru stayed in Celagi, and both individuals were now observed robbing at higher rates per observation hour (Gru: 1.29, Hanoman: 1.31). If individual levels of robbing and bartering frequency can change so drastically in just over a year, then we should expect to see differences in robbing and bartering practices within a single group over a time period of nearly eight years, which is the approximate gap between data collection in this study and the original published account for this population^1^. Thus, robbing and bartering practices for each group are likely to vary over time as group composition and individual robbing propensities change. Such dynamism at the individual level, and its relation to group-level outcomes, lends further credence to the suggestion that robbing and bartering is a cultural behavior with socially influenced exhibition patterns^1,4^.

## Conclusions

This study focused on naturalistic observations of spontaneous robbing and bartering in free-ranging subadult male long-tailed macaques. Like previous work on this topic, we found that individuals vary in their propensity to engage in robbing and bartering, but we offer the additional insight that, at least for the focal subadult males, the observed individual variation can, but does not necessarily, correlate with dominance status. More dominant subadult males were more efficient robbers than subordinate ones in one study group, but not the other. These results indicate that dominance status may have some effect on robbing and bartering, but that more complex variables, possibly associated with the specific patterns of social traditions/cultures and social structures within groups, are also at play. A major limitation to our study is the small sample size; therefore, future research with larger sample sizes is needed to more fully understand the factors associated with exhibiting robbing and bartering.

In focusing on the subadult males’ behavior and their relationships with one another, and comparing between group samples, our results suggest that the socially mediated, learned, and likely cultural behavior of robbing and bartering^1,2,4^ may be structured by both individual variation and intragroup social dynamics. Future work exploring the specific robbery strategies observed (e.g., direction of approach, target object), and to what degree individuals prefer one strategy over another would offer greater insight into the multiple components of this behavior. Furthermore, social network analyses amongst the subadult males at this site^17^ suggests it may be of interest to investigate the degree to which high social connectedness corresponds to a skillset associated with successfully developing the behavioral sequence of robbing and bartering, such as enhanced dexterity or an increased ability to navigate the requisite interspecies interactions in the exchange. Finally, tracking rejected exchange attempts, and which food rewards are offered in those instances, may further shed light on the potential valuation systems/processes macaques associate with food options or even the objects themselves.

## Data Availability

Data used in this study are available upon request from the corresponding author.
